# Investigation of the Weldability of 3D-Printed Multi-Material Materials (PLA and PLA Wood) Using Friction Stir Welding

**DOI:** 10.3390/polym16233249

**Published:** 2024-11-22

**Authors:** Gökhan Şahin, Nergizhan Anaç, Oğuz Koçar

**Affiliations:** Department of Mechanical Engineering, Faculty of Engineering, Zonguldak Bülent Ecevit University, Zonguldak 67100, Türkiye; sahingkhn.52@gmail.com (G.Ş.); oguz.kocar@yahoo.com.tr (O.K.)

**Keywords:** 3D printing, polymer, multi-material, friction stir welding

## Abstract

In the industry sector, it is very common to have different types of dissimilar materials on the same construction rather than products made from a single type of material. Traditional methods (welding, mechanical fastening, and adhesive bonding) and hybrid techniques (friction stir welding, weld bonding, and laser welding) are used in the assembly or joining of these materials. However, while joining similar types of materials is relatively easy, the process becomes more challenging when joining dissimilar materials due to the structure and properties of the materials involved. In recent years, additive manufacturing and 3D printing have revolutionized the manufacturing landscape and have provided great opportunities for the production of polymer-based multi-materials. However, developments in the joining of multi-material parts are limited, and their limits are not yet clear. This study focuses on the joining of 3D-printed products made from PLA-based multiple materials (PLA and PLA Wood) using friction stir welding. Single-material and multi-material parts (with 100% infill ratio and three different combinations of 50% PLA/50% PLA Wood) were welded at a feed rate of 20 mm/min and three different tool rotational speeds (1750, 2000, and 2250 rpm). Tensile and bending tests were conducted on the welded samples, and temperature measurements were taken. The fractured surfaces of the samples were examined to perform a damage analysis. It is determined that the weld strength of multi-materials changes depending on the combination of the material (material design). For multi-materials, a welding efficiency of 74.3% was achieved for tensile strength and 142.68% for bending load.

## 1. Introduction

The process of printing parts with a 3D printer has become one of the most popular additive manufacturing methods. Thanks to the developments in 3D printer technologies, multi-material components can be produced using more than one material and a single or more nozzles in the same production process [1,2]. In multi-material structures, the ability to change materials in various combinations enhances the properties of the products. Although it is recommended, if possible, to use the same base material (either by using materials of different colors or by using composite combinations with a shared matrix) in the production of multi-material parts with a 3D printer [3], researchers have worked with various groups of materials. These material groups vary between poly (lactic acid)–thermoplastic polyurethane (PLA–TPU) [4], acrylonitrile butadiene styrene–polylactic acid (ABS–PLA), acrylonitrile butadiene styrene–thermoplastic copolyesters (ABS–TPC) [5], TPU–PLA configuration/form [6], PLA–PET (polyethylene terephthalate), etc. There may be no need for joining or assembly processes in multi-material structures. The number of parts and production time are reduced with the ability to produce complex forms [4]. However, there are some limitations to multi-material production. The compatibility of the materials used in the product and the adhesion between the contact surfaces of the materials can impact the production process [4,6,7]. For these reasons, joining multi-materials requires special care. Traditional methods commonly used for joining materials can also be applied to parts produced by 3D printing. In a recently published study, adhesive bonding was used to join structures made from multi-materials [8].

In the literature, friction stir welding [9,10,11] and adhesive bonding applications [12,13,14,15,16] are frequently reported for joining plastics produced using 3D printers. However, joining multi-material parts has not received sufficient attention from researchers. In the literature review, no information was found on the use of friction stir welding in joining multi-materials. The superior strength conferred by the welding process in comparison to adhesive bonding has led the authors of this study to advocate for the consideration of friction stir welding as a viable method for joining multi-materials. Friction stir welding is one of the solid-state methods first used in joining aluminum materials. Since the introduction of FSW, its application area has expanded considerably, being used in the welding of other metals and polymer materials.

Koçar et al. conducted an experimental study on the joining of 3D printed PLA Wood plates by friction stir welding using three different feed rates (20, 40, and 60 mm/min), three different tool rotational speeds (1250, 1750, and 2250 rpm), and three different pin geometries (triangle, square, and screw). After determining the highest welding strengths obtained for each pin geometry in the study, the weldability of PLA Wood material with PLA–CF and PLA Plus materials was investigated using these parameters. The results showed that the highest welding quality in joining PLA Wood plates was obtained with 74.5% efficiency using square pin geometry, 20 mm/min, and 1750 rpm process parameters. It was stated that PLA Wood plates were successfully joined with PLA–CF and PLA Plus materials with the same process parameters (square pin, 20 mm/min, and 1750 rpm) [10].

In another study, the weldability of PLA plates at 20, 30, and 40 mm/min feed rates, 700, 1400, and 2000 rpm tool rotational speeds, and cylindrical, threaded, and conical pin geometries was investigated. As a result, weak joining strength was obtained at 700 and 2000 rpm, and the best weld strength was obtained at 1400 rpm tool rotational speed. High tool feed rates caused low strength. As a result, the highest weld strength was obtained using a cylindrical pin geometry. The temperatures occurring in the welding process where the highest weld strength was obtained were measured between 75–110 °C [17].

This study focuses on the investigation of the changes in mechanical properties in the joining of PLA-based multi-materials (PLA and PLA Wood) by friction stir welding. For this purpose, single-material and multi-material parts with a 100% infill ratio (in three different combinations as 50% PLA/50% PLA Wood) were joined by SKK at 20 mm/min feed rate and three different tool rotational speeds (1750, 2000, and 2250 rpm). Tensile and bending performances were investigated after welding. Weld defects were determined by visual inspection of the weld area. Finally, the relationship between the heat values generated during welding and the weld strength was discussed.

## 2. Material and Methods

### 2.1. Materials

In the study, two different thermoplastics with a diameter of 1.75 mm were used: PLA (polylactic acid) and wood-added PLA (PLA Wood). PLA filaments are the most commonly used filament type in 3D printers. PLA filaments are thermoplastics that are non-toxic, biodegradable, and harmless to human health because they are produced from organic materials such as corn starch and sugar cane, as well as having advantages such as ease of printing and production [18,19]. PLA Wood filament is a composite type of PLA that is reinforced with 30% wood. Wood is a biodegradable material that is commonly found in the form of wood residues. Small wood pieces are ground into finer wood dust, allowing them to be used as a filler material alongside traditional plastics [20].

The incorporation of real wood reinforcement into PLA results in enhanced temperature resistance, reduced visibility of layer lines, and a matte wood appearance [21]. The wood additive imparts a distinctive color to the filament, providing an attractive finish for decorative prints. The properties of the filaments used in this study are presented in Table 1.

### 2.2. Printing Samples from 3D Printer

**Figure 1:**
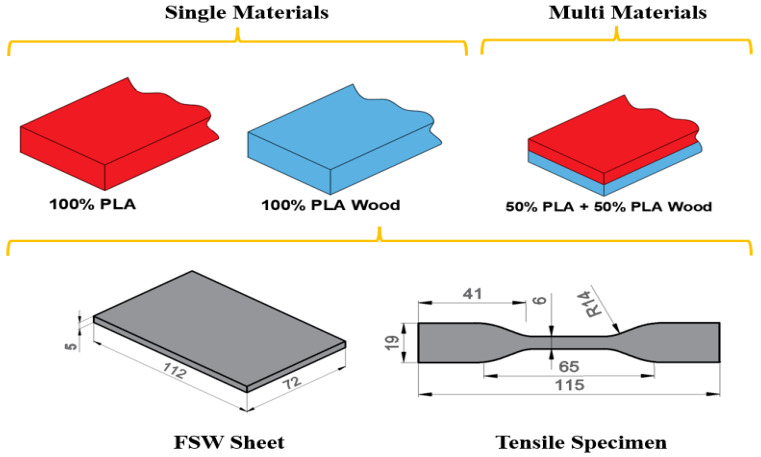
Single and multi-materials.

All the samples in the study were printed using Ender 3 S-1 (Creality, Shenzhen, China [24]) as single materials and multi-parts (50% PLA and 50% PLA Wood), at a 100% infill ratio, as flat in the XYZ plane, with a linear pattern, and in (45°/−45°) stacking order. Plates for FSW were prepared with dimensions of 112 × 72 × 5 mm. The layer thickness is fixed at 0.2 mm in all samples. In Figure 1, the dimensions of the tensile test specimens (ASTM D638-10 [25]) used to determine the infill ratios used in single and multi-materials, the FSW plate, and the mechanical properties are given.

The materials and printing parameters used directly affect the quality and mechanical properties of the 3D product [26,27]. Printing parameters were determined using the values recommended by the manufacturer and previous studies [28,29]. Printing parameters for PLA and PLA Wood are given in Table 2.

### 2.3. Experimental Design

Weld quality in the FSW process is affected by many variables. The most important of these variables are material type, tool feed rate, shoulder, pin geometry, tool rotational speed, plunging speed, and inclination angle [30,31,32,33]. Therefore, it is very important to determine the process parameters correctly. However, today, FSW process parameters are determined by the trial-and-error method. When the literature is examined, it is seen that in the studies on joining PLA using FSW, the process parameters used are a tool feed rate of 3–40 mm/min, a tool rotational speed between 600–2000 rpm, and pin geometry as cylindrical, screw, truncated taper, and tapered [34,35,36,37]. The authors of this study used three different feed rates (20, 40, and 60 mm/min), three different tool rotational speeds (1250, 1750, and 2250 rpm), and three different pin geometries in their previous paper on joining sheets prepared from PLA Wood filament. In their studies conducted on PLA Wood plates, the best result was obtained with 74.51% efficiency at 20 mm/min feed rate, 1750 rpm tool rotational speed, and square pin geometry [10]. As a result of the general evaluation, square pin geometry, a tool feed rate of 20 mm/min, and three different tool rotational speeds (1750, 2000, and 2250 rpm) were selected for joining different combinations of multi-materials using FSW. Table 3 shows the parameters determined for joining single and multi-materials with FSW.

### 2.4. Friction Stir Welding Parameters

In FSW, the tool consists of two parts: the shoulder and the pin. Since the shoulder part of the FSW tool rubs against the surfaces of both parts to be joined, it plays an important role in obtaining the friction heat, which is critical in friction stir welding [38]. By covering the weld seam from the top, it restricts the movement of the material that becomes plasticized during welding and moves upwards due to the rotation of the pin; thus, the material is pushed downwards by the shoulder and remains in the weld area. The FSW tool is made of AISI 1040 steel (Sağlam Metal, Kocaeli, Türkiye). The FSW tool shoulder diameter (20 mm), shoulder height (15 mm), shoulder plunging depth (1 mm), tool plunging depth (4.5 mm), and pin geometry are kept constant as square.

**Figure 2:**
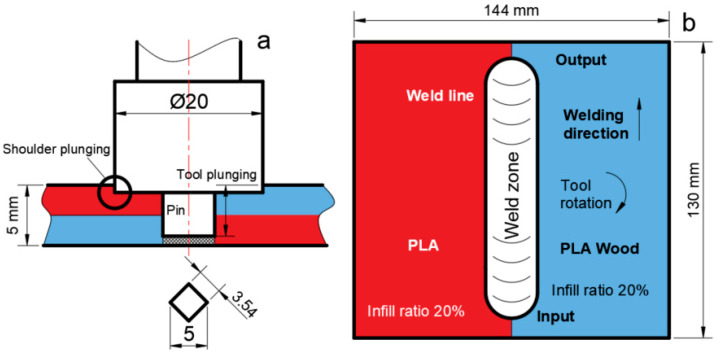
(**a**) FSW tool dimensions, penetration distance, (**b**) tool input/output points, tool feed rate, and rotation directions.

In order to prevent the plates from moving during the FSW process, a special mold and appropriate fastening elements are used, because in the FSW process, the pin forces the plates to separate from each other. Therefore, a stable connection should be made using the mold and fasteners. After the sheets were fastened to the fixed, the FSW tool approached the input point from the top of the sheets with a feed rate (Z axis) of 15 mm/min. The tool was kept for 10 s after reaching the plunging depth. Figure 2 shows the FSW tool dimensions, tool plunge distance, input points, output points, tool rotation direction, and welding direction.

### 2.5. Determination of Mechanical Properties

A 5 kN capacity WDW-5 model universal tensile tester (Jinan Chenda Testing Machine Manufacturing Co., Ltd., Jinan, Shandong, China) was used to determine the weld strength of the base material and friction stir welded plates. Tensile tests were carried out at room temperature and 2 mm/min tensile speed. In addition, each test was repeated 3 times, and the averages were taken. Weld strengths were evaluated according to the tensile test results. The durometer (Shore D) hardness measurement method was used in hardness measurements. Hardness measurements were made according to ASTM D2240-15 [39]. Hardness measurements were taken from the base material, the heat-affected zone, and the weld zone in at least five replicates. The microstructure of the weld area was examined using a digital microscope. Finally, thermal images were taken with a Fluke Ti32 thermal camera (Fluke Corporation, Everett, WA, USA) to investigate the effects of tool geometry, feed rate, and rotational speed on heat generation during the FSW process.

## 3. Results and Discussion

This study used two different filament materials (PLA and PLA Wood). Both single-material plates and multi-material plates were produced from the filaments. The sheets were printed with a 100% infill ratio and a layer thickness of 0.2 mm. Multi-materials consist of 50% PLA and 50% PLA Wood. The prepared plates were joined using friction stir welding at a feed rate of 20 mm/min and three different tool rotational speeds (1750, 2000, and 2250 rpm) in various combinations. The weldability of the multi-materials using the FSW method was investigated. The weld strengths of the joints were evaluated, and the optimal processing parameters were determined. Finally, the welding defects that occurred in the welded samples were examined by taking images from the weld area.

### 3.1. Mechanical Properties of Base Materials

**Figure 3:**
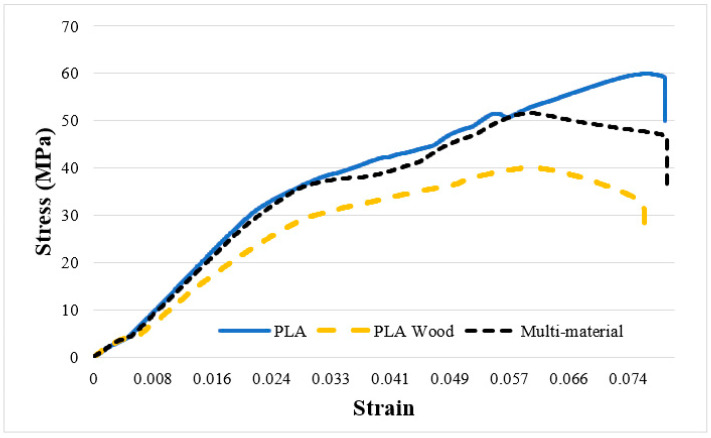
PLA, PLA Wood, and multi-material stress–strain graph.

**Figure 4:**
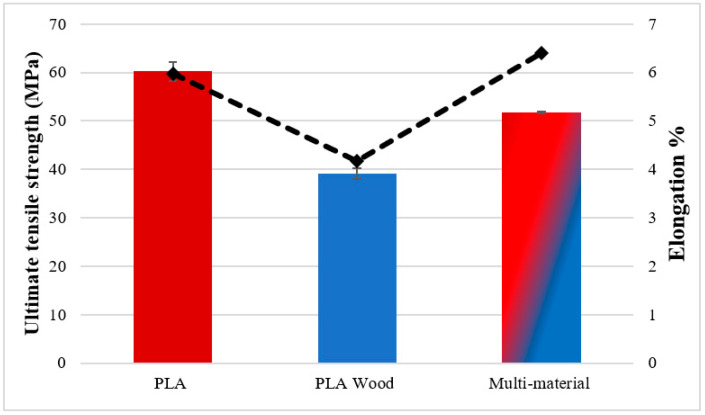
UTS and percentage elongation values of base materials.

To determine the mechanical properties of the base materials (PLA, PLA Wood, and multi-material), tensile tests (Figure 3) and hardness measurements were conducted. The tensile test results showed that the tensile strengths were 60.2 ± 1.95 MPa for PLA, 39.12 ± 1.17 MPa for PLA Wood, and 51.71 ± 0.20 MPa for the multi-material. The ultimate tensile strength (UTS) values of the base materials are represented by red and blue columns in Figure 4, while the percentage elongation values are shown as a dashed line. It can be observed that PLA Wood has the lowest UTS and elongation values. The decrease in the mechanical properties of PLA Wood compared to PLA is due to the negative impact of the wood additive on adhesion between layers and the irregularities created by the wood additive [10,40]. In the multi-material, there was an improvement of 132% (for UTS) and 153% (for elongation) in UTS (51.71 ± 0.20 MPa) and elongation values (6.4 ± 0.18) compared to PLA Wood, respectively. It can be said that this situation in multi-materials is due to the good adhesion of the layers in the material transition region (contact surfaces/interface) of PLA and PLA Wood. Shore D hardness values were measured as 87.25 ± 0.95 for PLA and 72.25 ± 0.25 for PLA Wood.

**Figure 5:**
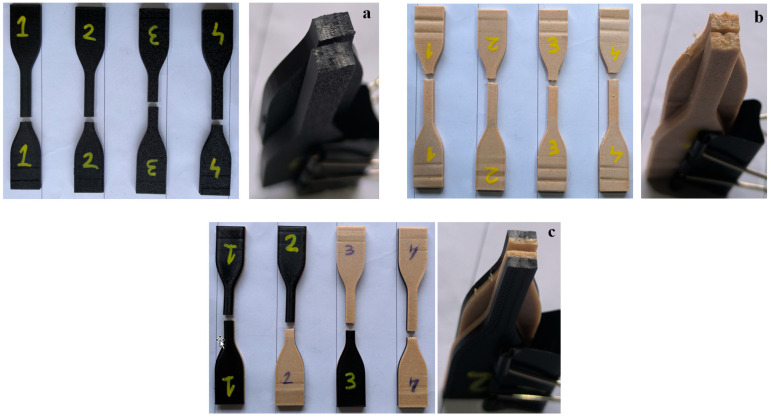
Fracture surfaces of PLA (**a**), PLA Wood (**b**), and multi-material (**c**) after tensile testing.

The images of the fracture surfaces of the base materials (for single-material and multi-material samples) after the tensile testing are given in Figure 5. The flat and sharp fracture shape of the PLA samples in Figure 5a shows that the tensile load follows the angle of the layer lines forming the structure of the samples [41]. In Figure 5b, the rupture in PLA Wood samples occurred on the surface perpendicular to the tensile force, and the fracture shape was indented like a reciprocal tooth mechanism. In addition, extensions that are thought to be wood particles in the filament are observed at the fracture ends of PLA Wood [40]. The fracture type of the multi-material tensile samples given in Figure 5c is similar to the fracture types of the PLA and PLA Wood materials that make up these samples (Figure 5a,b). This situation shows that the tensile load is distributed uniformly in the multi-material samples. In addition, it was observed that the multi-material tensile samples did not separate from the interface after the tensile testing. This shows that PLA and PLA Wood are compatible materials in multi-material product design and proves that their selection as multi-material was made appropriately.

**Figure 6:**
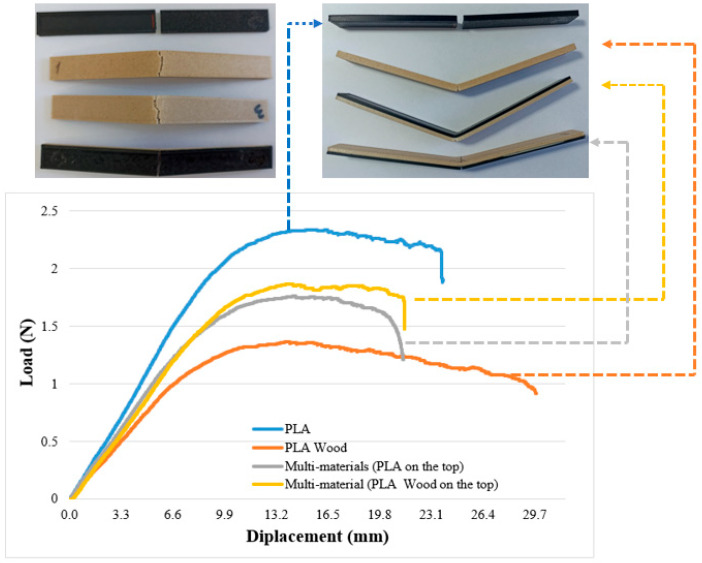
Three-point bending test graph.

Figure 6 presents the three-point bending test graphs for the base materials. The samples prepared for the bending tests include PLA, PLA Wood, and multi-material samples. According to the three-point bending test results, the maximum bending load was determined as 103.32 ±2.5 N for PLA, 61.45 ± 1.25 N for PLA Wood, 75.81 ± 0.21 N for multi-material (PLA on top), and 81.42 ± 2.70 N for multi-material (PLA Wood on top). The highest bending load was found in PLA, while the bending loads of multi-materials were between the bending loads of PLA and PLA Wood. In multi-materials, it was determined that the material position affected the bending load. In the bending test, PLA samples broke from the point where the force was applied. In PLA Wood and multi-materials, the fracture started with a crack in the lower surface of the part, but the samples did not break completely.

### 3.2. Visual Inspection of Welding Area

**Figure 7:**
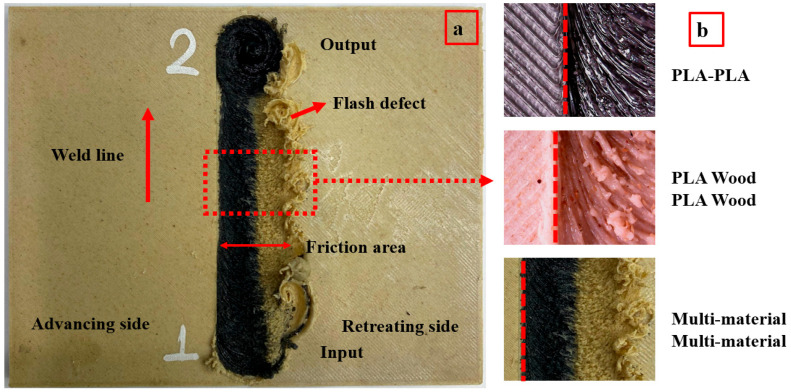
(**a**) FSW basic concepts, (**b**) FSW weld area appearance (The dashed lines show the boundary separating the weld zone and the material. 1: input point, 2: output point).

Figure 7a shows the appearance of the weld area and basic concepts in the parts welded using FSW. When the figure is examined, entry and exit points, weld line, tool rotation direction, advancing side (AS), retreating side (RS), and flash defects are seen. After the parts were fixed so that they did not move, the FSW tool plunged 4.5 mm at 15 mm/min from the input point. The tool was kept at the entry point for 10 s to generate the necessary heat before welding, and the welding process was performed with a feed rate (Z axis) of 20 mm/min. It was noted that the appearance of the weld lines between the RS and AS (advancing side) varied based on the material flow (mixing quality). Figure 7b shows images of the weld zone after FSW, where the flow traces of the material in the weld zone can be seen clearly.

**Figure 8:**
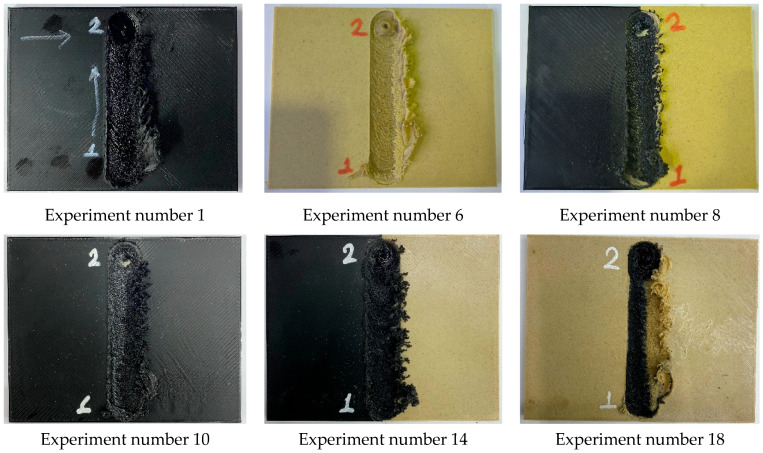
Images of some welded samples.

Figure 8 displays images of some samples after FSW. In all samples in the experiment, the formation of flash was observed on the retreating side. Flash occurred relatively more frequently at the tool input point. The reason for this is that the tool is left for 10 s to generate the necessary heat at the input point. It can be stated that the heat generated while keeping the tool made the material relatively more fluid, causing it to be pushed outward as the tool advanced [42]. Another defect encountered in friction stir welding is the keyhole defect that occurs at the tool entry (keyhole) and exit (exit hole) [43]. No keyhole defect was observed in this study. It was determined that exit hole defects occurred in experiments 4, 5, 6, 7, 8, 9, 13, 16, and 18. The pairs where exit hole defects did not occur were PLA–PLA and multi-material samples where PLA was on top. This can be explained by PLA being more fluid and the material around the tool filling the gap during exit.

**Figure 9:**
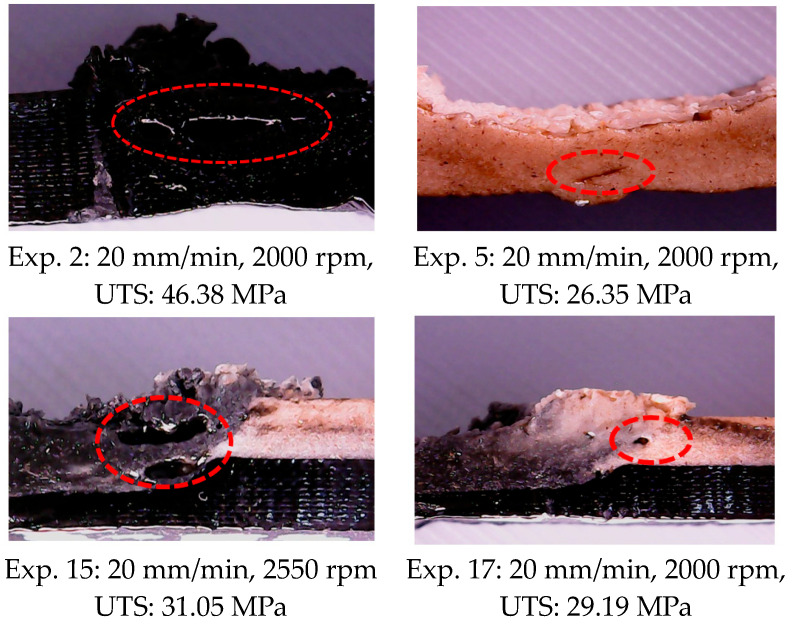
Some of the samples with tunneling defects (The red circles mark the areas where there are weld defects).

Images of some of the samples with tunneling defects in the examinations performed after FSW are given in Figure 9. The tunneling defect is a type of defect that is frequently seen in FSW and negatively affects weld strength [44]. The tunneling defect occurs due to incorrect selection of FSW process parameters, resulting in insufficient heat generation, insufficient plasticization of the material in the weld zone, and unbalance in material movement around the pin [45]. The strength value of the material decreases as the crack formation starts from the tunneling defect area in the material exposed to the load. In this study, tunneling defects were determined in experiments numbered 2, 5, 15, and 17. While tunneling defects occurred inside the weld area in experiment 2 and experiment 5, they occurred in the area where the tip of the tool shoulder touched in experiment 15 and experiment 17. It is thought that this situation is due to poor material flow in experiment 2 and experiment 5 and to the outward flow of the plasticized material in experiment 15 and experiment 17.

**Figure 10:**
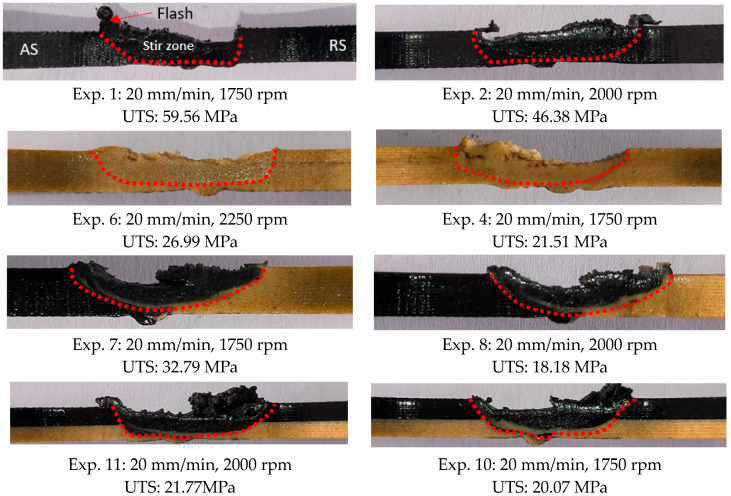

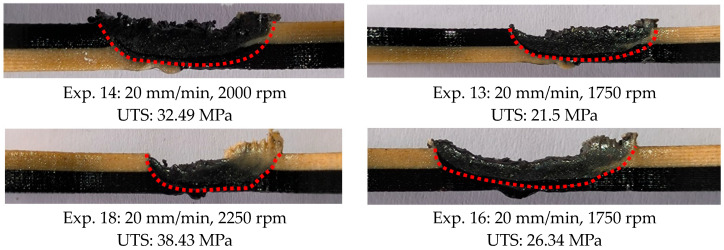
Images of welded areas (AS: advancing side, RS: retreating side The red lines show the boundary separating the weld zone and the material).

Figure 10 shows images of the weld area of the samples with the best and worst UTS according to the tensile test results. In Figure 10, a basin-shaped stir zone was formed towards the accumulation side in samples with flash defects [46]. While material flow occurred in all samples, irregularities were observed in those with lower UTS. It is understood that optimizing the FSW process parameters is necessary to eliminate these defects.

**Figure 11:**
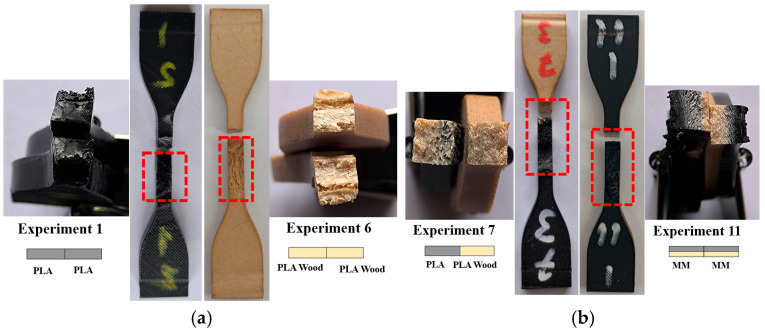

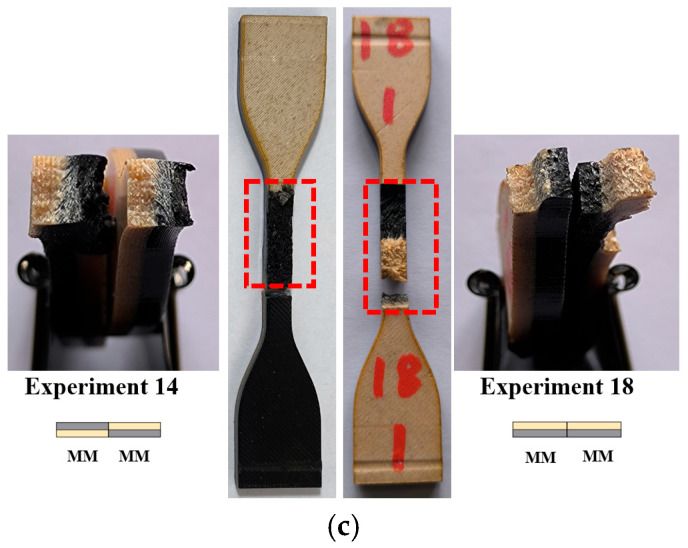
Fracture surfaces in tensile samples with best UTS after FSW (**a**) for a single material, (**b**) for dissimilar materials and multi-materials, (**c**) for multi-materials (The red box indicates the weld zone).

Figure 11 presents the fracture surfaces of the welded tensile samples with the best UTS. When comparing the rupture regions in Figure 11a with the fracture images of the base PLA and PLA Wood tensile samples shown in Figure 5a, it is evident that melting has occurred in the weld zones in Figure 11a, and the layers have fused together due to the melting and cooling process. The UTS value of experiment 7 was between the UTS values obtained in experiment 1 and experiment 6. In the welded sample of experiment 7, the fracture occurred on the PLA Wood side because the strength of the PLA material was higher than that of PLA Wood.

In the rupture surfaces of experiment 11, experiment 14, and experiment 18 (Figure 11b,c) visual examinations revealed the presence of a material transition zone with a similar color. In the welded samples produced from multi-materials, the color change occurring at the interface between PLA and PLA Wood materials is distinctly visible. Additionally, experiment 14 and experiment 18 were produced as multi-material samples (with different material combinations), while experiment 7 samples were created using two different materials (PLA and PLA Wood). When comparing these test samples, it was found that using PLA Wood in combination with PLA (PLA–PLA Wood) is more advantageous than using PLA Wood alone (PLA Wood–PLA Wood). Specifically, having PLA positioned underneath contributes to increased weld strength. During mixing (the material movement caused by tool rotation), the material on the top is pushed outward due to the effects of plasticization, resulting in flash defects. Thus, a decrease occurs in the material located on the top of the weld area. Therefore, in the multi-materials where PLA Wood is positioned on top, it is thought that the PLA Wood flows out of the weld zone (resulting in flash formation), while the PLA remaining underneath contributes to increasing the joint strength. The higher UTS value of the PLA material compared to PLA Wood had a positive effect on welding strength. At the same time, the fact that there was no filler material in PLA as in PLA Wood also increased this effect.

### 3.3. Result of FSW Strength

Table 4 shows the tensile strengths and welding efficiency (%) of PLA, PLA Wood, and multi-material parts joined using square pin geometry, at 1750, 2000, and 2250 rpm rotational speed, and at 20 mm/min feed rate. Welding efficiency (%) was calculated separately for each experiment according to the weld strengths obtained after welding and the UTS values obtained from the reference samples and added to Table 4. The first three rows in Table 4 show the UTS values of the reference samples. The highest weld strengths were found to be 59.56 MPa (experiment 1) for PLA–PLA at 1750 rpm tool rotational speed, 26.99 MPa (experiment 6) for PLA Wood–PLA Wood at 2250 rpm tool rotational speed, 32.79 MPa (experiment 7) for PLA–PLA Wood at 1750 rpm tool rotational speed, and 38.43 MPa (experiment 18) for multi-material at 2250 rpm tool rotational speed.

**Figure 12:**
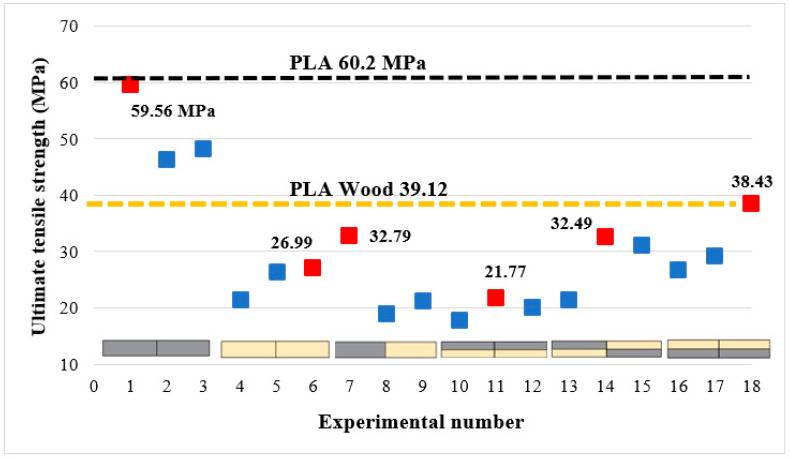
Tensile strengths of the welded samples.

In the joining of multi-materials using FSW (experiments 10–18), it was observed that the weld strength varied depending on the material placement. In experiment 10, experiment 11, and experiment 12, the upper side of the multi-material was PLA, and the welding strength efficiency was limited to 34–42%. Since PLA was in the area where the shoulder contacted the part, flash formation occurred by flowing outwards during welding. Figure 12 shows the UTS values of the samples after FSW. The red dots on the graph show the highest tensile strength of each material group. In addition, the tensile strengths of the base materials (black line: PLA, orange line: PLA Wood) are added. When the mechanical properties of the base materials were examined, it was determined that the mechanical properties of PLA were better than those of PLA Wood. In their studies on the comparison of the mechanical and thermal conductivity properties of filament materials, Furkan et al. stated that the continuity of the layers was disrupted during printing due to the wood filler material and that the wood additive negatively affected the adhesion between the layers [40]. While the wood additive gives the filament a nice appearance and a wood smell during printing, it negatively affects the mechanical properties. When evaluating Figure 12, experiments 1, 2, and 3 should be taken into account according to the mechanical properties of PLA, and experiments 4–18 should be taken into account according to the mechanical properties of PLA Wood, since in welded joints, the part with the lowest strength determines the weld strength.

In the experiments where PLA–PLA plates were joined (experiments 1–3), the highest strength (59.56 MPa) was obtained at a tool rotational speed of 1750 rpm. A decrease in weld strength occurred with the increase in tool rotational speed. This situation can be explained by the fact that high tool rotational speed at low feed rates increases the friction between the tool and the part and because of the excess heat generated during welding. It is thought that the welding strength is negatively affected because excessive heat in the welding area increases plasticity.

In the joining of PLA Wood–PLA Wood plates (experiments 4–6), the highest strength (26.99 MPa) was obtained at a tool rotational speed of 2250 rpm. Koçar et al. investigated the effects of pin geometry, tool feed rate, and tool rotational speed on the weld strength in the joining of PLA Wood plates using FSW. In their study, they determined the best weld strength as 28.18 MPa at square pin geometry, a 20 mm/min feed rate, and a 1750 rpm tool rotational speed [10]. The results of that study were close to the ones obtained in the present work. However, it can be said that the wood additive in PLA Wood negatively affects both 3D printing and friction stir welding. In PLA–PLA Wood plates (experiments 7, 8, and 9), the best weld strength was obtained with 83.82% efficiency at a 1750 rpm tool rotational speed. In experiments 7, 8, and 9, an increase in tool rotational speed led to a decrease in weld strength. It has been observed that lower tool rotational speeds should be preferred at low feed rates when joining PLA and PLA Wood plates.

In experiments 10, 11, and 12, where PLA was positioned on top, the weld strength of the multi-materials significantly decreased. This can be attributed to the fact that PLA was situated on the upper side, leading to the material being pushed outward during the FSW process. In experiments 13, 14, and 15, the repositioning of PLA positively impacted the weld strength. In experiments 16, 17, and 18, where PLA was placed at the bottom, a welding efficiency of 74.3% was achieved at a tool rotational speed of 2250 rpm. In multi-materials, higher weld strengths were achieved at tool rotational speeds of 2000 and 2250 rpm compared to a tool rotational speed of 1750 rpm. This can be attributed to the need for relatively high temperatures for effective joining in multi-materials.

### 3.4. Bending Test Results

The welding efficiency (%) values presented in Table 5 have been calculated based on the bending test results and reference samples. The first four rows in Table 5 contain the bending loads of the reference samples. In experiments numbered 10–18, two different reference samples were based on material design to achieve more reliable results in calculating welding efficiency (%). In the reference multi-material sample coded “MM 1”, PLA was positioned on the upper part, while in the multi-material sample coded “MM 2”, PLA Wood was placed on top. Welding efficiency (%) was calculated separately for each experiment based on the weld strengths obtained after welding and the bending load values obtained from the reference samples (PLA, PLA Wood, MM 1, and MM 2). These values have been added to Table 5.

The highest bending loads were found to be 145.36 N at a tool rotational speed of 1750 rpm for PLA–PLA (experiment 1), 47.27 N at a tool rotational speed of 2250 rpm for PLA Wood–PLA Wood (experiment 6), 62.99 N at a tool rotational speed of 2000 rpm for PLA–PLA Wood (experiment 8), and 108.17 MPa at a tool rotational speed of 1750 rpm for the multi-material (experiment 13).

**Figure 13:**
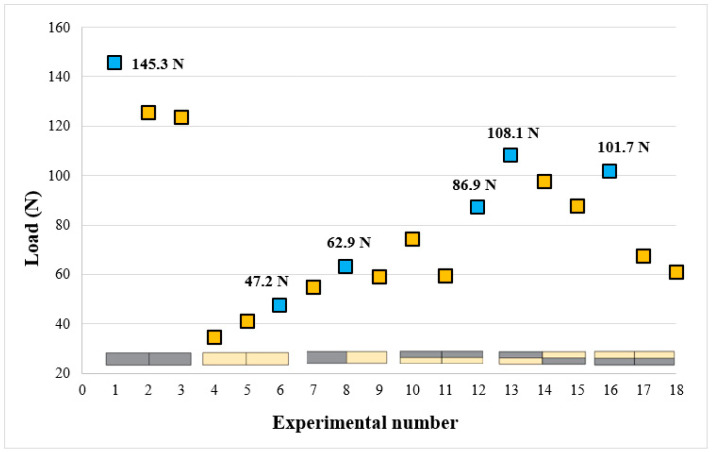
Bending test results of welded materials (Blue colors indicate the highest bending load value of each group).

Figure 13 presents the bending test results in a graphical format. The blue dots on the graph indicate the highest bending results for each material group. It was observed that an increase in the tool rotational speed negatively affected the bending load for the PLA–PLA combination, whereas an increase in the rotational speed enhanced the bending load for the PLA Wood–PLA Wood combination. This situation can be explained by the fact that in the PLA material group, the increasing temperature associated with the tool rotational speed leads to excessive plasticization of the material, negatively affecting the welding process. In contrast, for the PLA Wood material group, higher temperatures are required during welding to allow for the rejoining of the PLA and the additive. In the PLA–PLA Wood material pair, the highest bending load was achieved at 2000 rpm due to the increased proportion of PLA during the stirring process.

In multi-materials, the bending load was generally higher compared to the PLA, PLA Wood, and PLA–PLA Wood material pairs. In multi-materials with PLA on top, the bending load was measured at 86.95 N (114.69%), while in those with PLA Wood on top, it was 101.71 N (124.93%). For multi-materials using a cross arrangement, the bending load reached 108.17 N (142.68%). When the bending loads of multi-materials were compared, the highest bending load was determined as 108.17 N (142.68%) in the design where the materials were placed crosswise. According to the results obtained, it was determined that multi-materials had better performance in bending load after FSW. Especially when bending load is taken into consideration, cross-material design should be preferred.

### 3.5. Evaluation of Temperature Results

**Figure 14:**
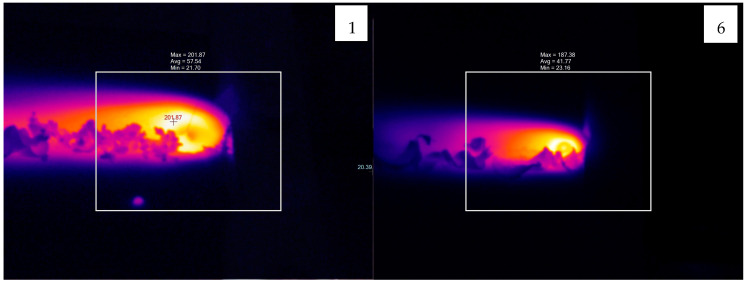

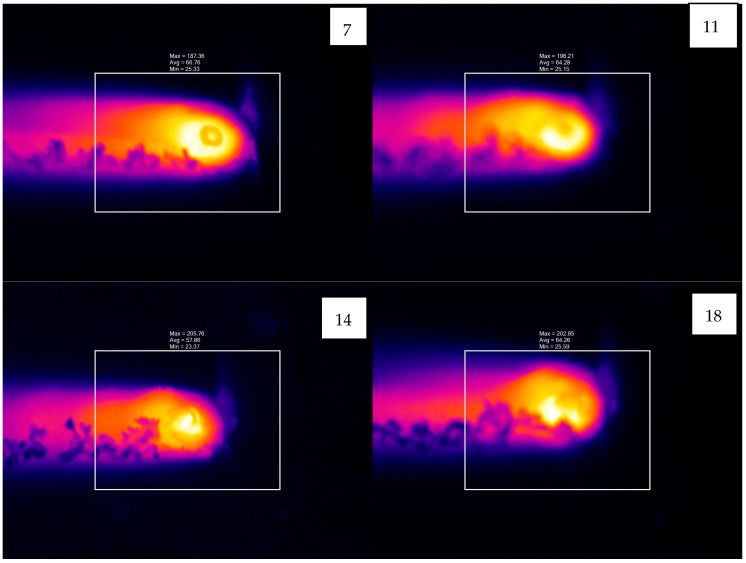
The thermal images of the samples with the best weld strength (experiments 1, 6, 7, 11, 14, and 18).

Figure 14 presents the thermal images taken for the highest weld strength obtained after FSW. During the FSW process, the temperature varied between 171.6 °C and 205.76 °C, depending on the tool rotational speed and the material pair. Table 5 shows the highest, lowest, and optimum temperatures recorded during welding (the temperature recorded at the best weld strength). The lowest temperatures recorded during the joining of PLA–PLA, PLA Wood–PLA Wood, and PLA–PLA Wood plates were 194.3 °C, 174.44 °C, and 171.6 °C, respectively, while the highest temperatures were 205.44 °C, 187.38 °C, and 194.16 °C, respectively.

The temperature difference between the highest and lowest values in PLA–PLA plates was 11.14 °C, in PLA Wood–PLA Wood plates it was 12.94 °C, and in PLA–PLA Wood plates it was 22.56 °C. This variation can be attributed to the random distribution of wood particles within the PLA Wood material. Randomly distributed wood particles cause inconsistencies in the friction between the tool and the material. In multi-material samples, the temperature differences between the highest and lowest values were 7.40 °C, 4.88 °C, and 8.55 °C.

It was observed that the heat generated during FSW was more balanced in multi-material samples. This can be attributed to the fact that the localized friction between the tool and the material is relatively more stable. In the multi-material samples, the highest UTS was measured in experiment 18, where the temperature value was 202.85 °C. The FSW parameters play a significant role in the heat generated during the welding process. This study has demonstrated that the type of different materials and multi-materials being joined significantly influences the friction between the tool and the workpiece, resulting in variations in the heat generated during the welding process. This factor should be considered, particularly when optimizing welding parameters for multi-materials.

Table 5 shows that the temperatures at which the highest weld strength was achieved for the material pairs of PLA–PLA, PLA Wood–PLA Wood, and PLA–PLA Wood fall between the minimum and maximum temperature values. The literature indicates that the best weld quality is achieved at optimal temperatures [17]. This is attributed to the fact that low temperatures adversely affect material flow, while high temperatures can lead to excessive plasticization of the material in the weld zone, negatively impacting strength [47].

**Figure 15:**
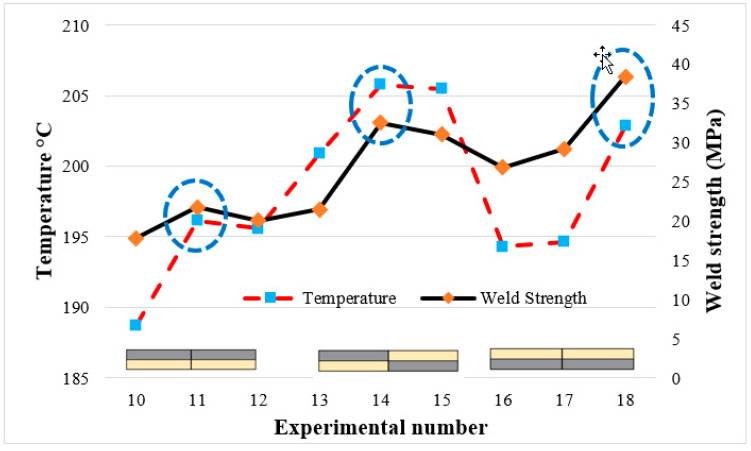
Temperature and weld strength in the joining of multi-materials.

Figure 15 presents the temperature values and weld strengths observed during the joining of multi-materials using FSW. Unlike the combinations of PLA–PLA, PLA Wood–PLA Wood, and PLA–PLA Wood, it has been determined that the best weld quality in multi-materials was achieved at the highest temperatures.

When examining the samples that provided the values presented in Table 6 (through visual inspection), it was observed that in the case of multi-materials, when the optimum temperature exceeded 196.12 °C (20 mm/min at 2000 rpm), the surface formed in the weld zone was very rough. Similarly, when the optimum temperature was below 196.12 °C, it was observed that the surfaces formed in the weld zone were relatively smooth, and the weld lines were more uniform.

## 4. Conclusions

In this study, different combinations of PLA and PLA Wood materials were joined using the FSW method at a feed rate of 20 mm/min and tool rotational speeds of 1750, 2000, and 2250 rpm. To evaluate the weld strength, the welded specimens were subjected to tensile and bending tests. The results obtained are summarized below.

In FSW, it was observed that inappropriate process parameters resulted in tunneling defects, which negatively impacted the weld strength. Additionally, exit holes and flash defects, commonly encountered in FSW, were identified in all samples. These defects highlight the necessity of optimizing the process parameters used in FSW to ensure better weld quality.

After the tensile test, the highest welding efficiency in multi-materials was found to be 74.3% at a feed rate of 20 mm/min and a tool rotational speed of 2250 rpm.

After the bending test, the highest welding efficiency in multi-materials was found to be 142.68% at a feed rate of 20 mm/min and a tool rotational speed of 1750 rpm. When the bending and tensile test results were compared, the welding efficiency obtained from the bending tests proved to be higher than the welding efficiency obtained from the tensile test.

Considering the test results, it was observed that the arrangement of materials in multi-materials significantly impacted the results. It was determined that the lowest and highest temperature differences resulting from process parameters in multi-materials were lower compared to the material pairs of PLA, PLA Wood, and PLA/PLA Wood.

## Figures and Tables

**Table 1:** Technical specifications of filament materials.

Mechanical properties	PLA [22]	PLA Wood [23]
Diameter (mm)	1.75	1.75
Brand	Filameon	Filameon
Color	Black	Light Brown
Tensile strength (MPa)	53	47
Elongation at break (%)	6	-
Density (g/cm^3^)	1.24	1.13

**Table 2:** Printing parameters.

Material	PLA	PLA Wood
Infill ratio (%)	100	100
Printing temperature (°C)	205	220
Build plate temperature (°C)	60	65
Print speed (mm/s)	60	45

**Table 3:** Parameters used in joining single and multi-materials.

Material	Material	FSW	Feed Rate (mm/min)	Rotational Speed (rpm)
PLA	PLA	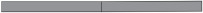	20	1750
PLA Wood	PLA Wood	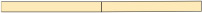		
PLA	PLA Wood	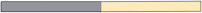		2000
Multi-material	Multi-material	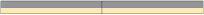		
		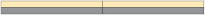		2250
		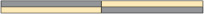		

PLA: Black color material, PLA Wood: Light brown material.

**Table 4:** Weld strengths and welding efficiency after FSW.

Exp.	Material	Material	Figural Representation	UTS (MPa)	FR (mm/min)	RS (rpm)	Welding Efficiency (%)		
							PLA	PLA Wood	MM
**Ref.**	PLA		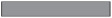	60.20 ± 1.95	--	--	--	--	--
**Ref.**	PLA Wood		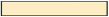	39.12 ± 1.17	--	--	--	--	--
**Ref.**	Multi-material		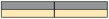	51.71 ± 3.4	--	--	--	--	--
**1**	PLA	PLA	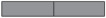	59.56 ± 2.40	20	1750	98.94	--	--
**2**	PLA	PLA		46.38 ± 4.60	20	2000	77.04	--	--
**3**	PLA	PLA		48.18 ± 3.30	20	2250	80.03	--	--
**4**	PLA Wood	PLA Wood	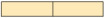	21.51 ± 0.10	20	1750	--	54.98	--
**5**	PLA Wood	PLA Wood		26.35 ± 4.40	20	2000	--	67.36	--
**6**	PLA Wood	PLA Wood		26.99 ± 0.40	20	2250	--	68.99	--
**7**	PLA	PLA Wood	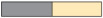	32.79 ± 2.70	20	1750	54.47	83.82	--
**8**	PLA	PLA Wood		18.88 ± 2.50	20	2000	31.36	48.26	--
**9**	PLA	PLA Wood		21.27 ± 2.40	20	2250	35.33	54.37	--
**10**	Multiple	Multiple	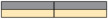	17.80 ± 1.80	20	1750	--	--	34.4
**11**	Multiple	Multiple		21.77 ± 1.30	20	2000	--	--	41.9
**12**	Multiple	Multiple		20.07 ± 1.20	20	2250	--	--	38.8
**13**	Multiple	Multiple	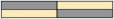	21.50 ± 1.40	20	1750	--	--	41.5
**14**	Multiple	Multiple		32.49 ± 2.20	20	2000	--	--	62.8
**15**	Multiple	Multiple		31.05 ± 1.80	20	2250	--	--	60.0
**16**	Multiple	Multiple	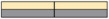	26.84 ± 4.10	20	1750	--	--	51.9
**17**	Multiple	Multiple		29.19 ± 2.90	20	2000	--	--	56.4
**18**	Multiple	Multiple		38.43 ± 4.30	20	2250	--	--	74.3

Exp.: experimental number, FR: feed rate, RS: rotational speed, Ref.: reference sample number, PLA: Black color material, PLA Wood: Light brown material.

**Table 5:** Bending test results and welding efficiency after FSW.

Exp.	Material	Figural Representation	Load (N)	FR (mm/min)	RS (rpm)	Welding Efficiency (%)			
						PLA	PLA Wood	MM 1	MM2
**Ref.**	PLA	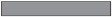	103.32 ± 2.5	--	--	--	--	--	--
**Ref.**	PLA Wood	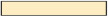	61.45 ± 1.25	--	--	--	--	--	--
**Ref.**	MM 1	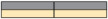	75.81 ± 0.21	--	--	--	--	--	--
**Ref.**	MM 2	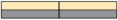	81.41 ± 2.7	--	--	--	--	--	--
**1**	PLA–PLA	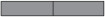	145.36 ± 5.3	20	1750	140.68	--	--	--
**2**			125.51 ± 8.2	20	2000	121.47	--	--	--
**3**			123.71 ± 0.7	20	2250	119.73	--	--	--
**4**	PLA Wood–PLA Wood	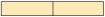	34.54 ± 1.3	20	1750	--	56.20	--	--
**5**			41.18 ± 6.0	20	2000	--	67.01	--	--
**6**			47.27 ± 4.7	20	2250	--	76.92	--	--
**7**	PLA–PLA Wood	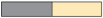	54.93 ± 1.5	20	1750	53.16	89.38	--	--
**8**			62.99 ± 5.8	20	2000	60.96	102.5	--	--
**9**			58.85 ± 1.1	20	2250	56.95	95.76	--	--
**10**	MM1–MM1	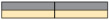	74.39 ± 7.9	20	1750	--	--	98.12	--
**11**			59.25 ± 2.1	20	2000	--	--	78.15	--
**12**			86.95 ± 1.0	20	2250	--	--	114.69	--
**13**	MM1–MM2	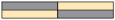	108.17 ± 2.5	20	1750	--	--	142.68	132.87
**14**			97.52 ± 4.6	20	2000	--	--	128.63	119.78
**15**			87.71 ± 0.4	20	2250	--	--	115.69	107.73
**16**	MM2–MM2	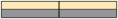	101.71 ± 1.9	20	1750	--	--	--	124.93
**17**			67.53 ± 0.4	20	2000	--	--	--	82.95
**18**			60.98 ± 5.9	20	2250	--	--	--	74.90

Ref.: reference sample number, PLA: Black color material, PLA Wood: Light brown material.

**Table 6:** Highest/lowest/optimum temperatures recorded during FSW.

Material Pair	Lowest Temperature (°C)	Highest Temperature (°C)	Temperature of Best Welding Strength (°C)	Temperature Difference (°C)
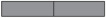	194.30 (Exp. 3)	205.44 (Exp. 2)	201.87 (Exp. 1)	11.14
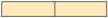	174.44 (Exp. 4)	187.38 (Exp. 6)	187.38 (Exp. 6)	12.94
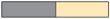	171.60 (Exp. 8)	194.16 (Exp. 9)	187.36 (Exp. 7)	22.56
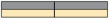	188.72 (Exp. 10)	196.12 (Exp. 11)	196.12 (Exp. 11)	7.40
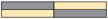	200.88 (Exp. 13)	205.76 (Exp. 14)	205.76 (Exp. 14)	4.88
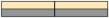	194.30 (Exp. 16)	202.85 (Exp. 18)	202.85 (Exp. 18)	8.55

PLA: Black color material, PLA Wood: Light brown material.

## Data Availability

Data are contained within the article.
